# Does Diagnostic Laparoscopy Still Have a Role in the Evaluation of Right Iliac Fossa Pain Versus Imaging Techniques or Experience?

**DOI:** 10.7759/cureus.30678

**Published:** 2022-10-25

**Authors:** Usman Rafique, Mohamed A Elfeky, Khalid Bhatti, Khurram Siddique

**Affiliations:** 1 General and Colorectal Surgery, The Royal Oldham Hospital, Northern Care Alliance NHS Foundation Trust, Oldham, GBR; 2 General Surgery, Salford Royal Hospital, Northern Care Alliance NHS Foundation Trust, Salford, GBR

**Keywords:** incidence of negative appendicitis, diagnosis of appendicitis, imaging in appendicitis, pain right iliac fossa, diagnostic laparoscopy, appendicitis treatment

## Abstract

Background

Right iliac fossa (RIF) pain is the most common cause of emergency surgical presentation, and a significant number of patients are eventually diagnosed with acute appendicitis. Typically, appendicitis is a clinical diagnosis, and it is prudent to take the patient to theatre for an early diagnostic laparoscopy (DL) to prevent complications as a result of acute appendicitis with the caveat it may lead to an increased number of negative appendectomies. The primary objective of this study was to ascertain the efficacy of DL in tackling RIF pain. The secondary objective was to compare the results among the diagnostic versus imaging groups (negative appendectomy rate, postoperative complications, and length of stay).

Methodology

The data were collected retrospectively for patients presenting with RIF pain in the surgical unit of the Royal Oldham Hospital between April 2017 and March 2019. The electronic database was utilized to identify all patients who underwent appendicectomy during this period. Patients were divided into two groups, group one had DL as their primary operation, and group two had imaging prior to surgery. Group two was further subdivided into computed tomography (CT) and ultrasonography (USG). Data included blood results, imaging reports, intraoperative findings, length of stay (LOS), postoperative complications, and histopathology results. The data were analysed using an Excel sheet and SPSS version 27 (IBM Corp., Armonk, NY, USA).

Results

A total of 340 patients were identified. Group one had 165 (48.53%) and group two had 175 (51.47%) patients. Most surgeries were carried out by middle-grade doctors (80.95%). Comparison with the histopathology report revealed that the negative appendectomy rate was 20% in group one, 3.8% in the CT group, and 27.5% in the USG group. The average length of stay was 2 ± 1.38 days in the diagnostic group and 3 ± 2.7 and 3 ± 0.8 days in subsequent groups.

Conclusions

This study shows that DL is a valuable first option when trained surgeons are available for tackling RIF pain, particularly in the young age group where it can reduce the risk of radiation exposure, decrease LOS, and avoid complications because of perforation.

## Introduction

Right iliac fossa (RIF) pain is almost always a surgical referral from the emergency department, and the most common differential diagnosis is acute appendicitis. Despite the major advancements in laboratory and imaging techniques, achieving 100% accuracy for acute appendicitis remains a surgical dilemma, and the selection of appropriate investigative tools is still questionable because of a lack of guidelines [1,2]. The most common diagnostic interventions after clinical assessment include ultrasonography (USG) of the pelvis, abdominopelvic computed tomography (CT) with intravenous contrast, and diagnostic laparoscopy (DL). The USG is a non-invasive method without the risk of radiation but is operator-dependent with variable success rates in diagnosing acute appendicitis [3]. Most studies looking at its efficacy have failed to establish consistent diagnostic specificity and sensitivity [4]. On the other hand, CT scan has shown approximately 98% sensitivity, but radiation exposure and nephrotoxicity limit its use in certain individual groups [5]. DL is a reasonably safe approach in dealing with RIF pain provided appropriately trained surgeons are available. In the United Kingdom, DL has a negative appendectomy rate of around 20% [6]. The focus of our study was to compare the accuracy of the patients undergoing DL as their primary procedure (group one) versus those who underwent imaging (group two) prior to the surgical intervention. The secondary objective was perioperative complications, length of stay (LOS), conversion to open, and level of the operating surgeon between the two groups.

## Materials and methods

**Figure 1:**
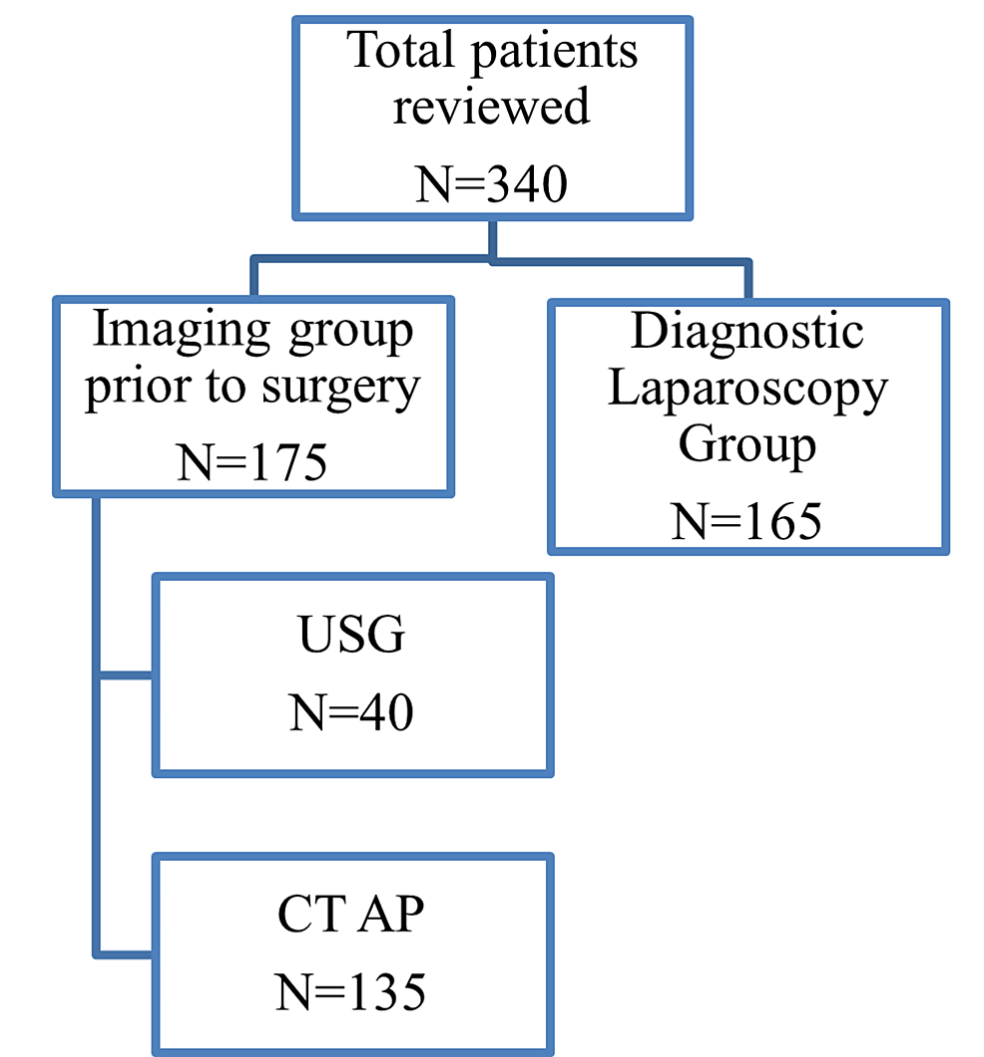
Flowchart of the study participants. CT AP: computed tomography abdominopelvic; USG: ultrasonography

This retrospective study was conducted at the Royal Oldham Hospital in the surgical department from April 2017 to March 2019. All patients who underwent appendicectomy in the study period were included. Patients with significant comorbidities, other causes of RIF pain, and open appendicectomy were excluded. Patients were searched in the hospital health view system using procedure-specific code and were divided into two groups based on the decision of the on-call surgical senior registrar and/or consultant. Group one underwent DL based on high clinical suspension of acute appendicitis and group two underwent preoperative imaging due to unclear diagnosis, suspected perforation, or suspected sinister pathology. The imaging group was further subdivided into the CT and USG group (Figure 1). This subdivision was based on the age and gender of the patients. USG was requested for young (less than 40) female patients, while the rest of the patients in the imaging group underwent CT. A proforma was designed for data collection that included age, gender, white cell count (WCC), C-reactive protein (CRP), procedure type, level of the operating surgeon, intraoperative findings, and LOS (Table 1). Histopathology was reviewed for every case (Table 2), and we also looked for postoperative complications, postoperative imaging, readmission, and the return to theatre. The data were anonymised and analysed using an Excel sheet and SPSS version 27 (IBM Corp., Armonk, NY, USA).

Categorical data were compared using appropriate statistics including the chi-square test and Kruskal-Wallis test, and the P-value of <0.05 was considered significant.

**Table 1:** Data regarding blood findings. WCC: white cell count; CRP: C-reactive protein; LOS: length of stay; CT: computed tomography; USG: ultrasonography; SD: standard deviation; IQR: interquartile range

	Diagnostic laparoscopy	Preoperative CT	Preoperative USG
Cases	165 (48.53%)	135 (39.71%)	40 (11.76%)
WCC (median ± SD)	14.0 ± 4.82	12.9 ± 4.6	12.6 ± 4.5
CRP (median ± SD)	24.8 ± 66.48	68 ± 112	16 ± 57
Median LOS	2 ± 1.138 (IQR = 2–3)	3 ± 2.7 (IQR = 2–3)	3 ± 0.8 (IQR = 2–3)
Conversion to open	2	5	0

**Table 2:** Histopathology results. CT: computed tomography; USG: ultrasonography; SD: standard deviation

Histopathology	Diagnostic laparoscopy	Preoperative CT	Preoperative USG
Normal appendix	33 (20%)	3 (2.22%)	11 (27.5%)
Appendicitis	132 (80%)	132 (97.78%)	29 (72.5%)
Total	165	135	40

## Results

A total of 340 patients were operated on during the study period. Out of these, 165 (48.53%) were included in group one (DL) while 175 (51.47%) in group two. In group two, CT was done in 135 (39.71%) and USG in 40 (11.76%) patients. The gender distribution was equal across all three groups with a p-value of 0.000, and the median age was approximately same in the DL and USG groups; however, CT group patients had a median age of 43 ± 15 (Table 3).

**Table 3:** Demographic data of the patients. CT: computed tomography; USG: ultrasonography; SD: standard deviation

	Diagnostic laparoscopy	Preoperative CT	Preoperative USG
Male	97	62	9
Female	68	73	31
Median age (years) ±SD	24 ± 9.75	43 ± 15.7	25 ± 11.9
Total	165	135	40

Group one had a negative appendicectomy rate of 20%. In this group, three patients had different pathological findings, including ovarian cyst, pelvic inflammatory disease (PID), and splenunculus with a normal-looking appendix but appendicectomy was performed as the appendix might be microscopically inflamed as well as to prevent diagnostic confusion in the future as those patients may represent with RIF pain which may cause a diagnostic dilemma. The average length of stay was 2 ± 1.138 days (IQR = 2-3) with the conversion from laparoscopy to open performed in only two cases. The Clavien-Dindo classification was used to describe postoperative complications [7] (Table 4). Most cases in DL were operated by middle-grade doctors (80.95%), and none of the patient’s had major postoperative complications or required theatre return in this group. Out of 33 normal appendicectomies, the operating surgeon successfully assessed the normal appendix during DL in 23 cases.

**Table 4:** Clavien-Dindo classification. USG: ultrasonography; SD: standard deviation

		Diagnostic laparoscopy	Preoperative CT	Preoperative USG
Cases		165	135	40
Clavien-Dindo classification	1	3	3	0
	2	4	8	3
	3	0	3	1

In group two, the accuracy of a CT scan for acute appendicitis was 96.2% with a specificity of 37.56%, a positive predictive value of 96.18%, and a negative predictive value of 75%. Only five discrepancies were noted on histology, three of them showed a normal appendix and two had a mucinous tumour on histopathology. On the other hand, 40 patients who had USG pelvis were taken into theatre for appendicectomy. Following the review of histopathology, only 29 (72.5%) had appendicitis, with a negative appendicectomy rate of 27.5%. The sensitivity and specificity of USG were 55.56% and 61.54%, respectively, and a positive predictive value of 75% and a negative predictive value of 40%. The LOS in these subgroups was 3 ± 2.7 and 3 ± 0.8 days, respectively.

## Discussion

The most common cause of acute abdomen presentation in UK hospitals is appendicitis, and it is estimated that 10% of the population will have acute appendicitis in their lifetime [8,9].

Making a correct diagnosis of appendicitis can be a conundrum, with 33% of atypical findings likely presenting as appendicitis; therefore, a combination of clinical and laboratory findings has been used to support the decision to operate [10,11]. The critical aspect is diagnosing appendicitis and operating as early as possible to avoid its complications which can significantly increase the length of hospital stay and the overall morbidity of patients [12].

In our series, group one went straight for DL and had good diagnostic accuracy based on clinical and laboratory findings. These patients had shorter hospital stay when compared with group two who underwent radiological investigations (CT, USG). This could be attributed to early decision-making for theatre and time saved from organizing CT and USG. The literature review supports the need for imaging prior to operating on older patients [13]. The negative appendectomy rate in the DL group was 20% which is comparable to other studies [8,12,14]. It is worth noting that 80.95% of the DL has been performed by appropriately trained middle-grade surgeons with no reported visceral injury which demonstrates that the procedure can be safely performed by a non-consultant with no major complications. It is worth noting that despite a normal-looking appendix in a few patients, they still had other types of pathologies such as ovarian cysts which could only be dealt with by means of laparoscopy.

In comparison, CT and USG are useful tools in aiding the diagnosis of acute appendicitis [15-17]. In many hospitals, imaging is essential in suspected acute appendicitis [18,19]. The use of a CT scan as an aid for diagnosing acute appendicitis is important, particularly in the older age groups where the differential diagnosis is broader [13]; however, there is a controversy regarding the routine use of the CT due to the hazards of ionizing radiation exposure and its overutilization of in the clear-cut presentation [20,21]. The CT scan of the abdomen may expose patients to what is equivalent to 400 Chest X-rays which would increase the risk of developing cancer or leukaemia. One advantage of CT is it can help decision-making in patients with equivocal clinical findings.

Hong et al. [22] compared the clinical assessment versus the use of the CT scan for diagnosing acute appendicitis and showed that the clinical assessment without a CT scan accurately identified patients who need to undergo surgery and shortened the period between examining the patients and going to surgery. They also suggested that the routine use of the CT scan should not be a standard of care in diagnosing acute appendicitis [22]. This supports the finding in our study which showed that patients who went directly for DL and had a good diagnostic yield had a shorter hospital stay when compared to patients who underwent preoperative image.

Furthermore, a USG scan in the RIF pain is the preferred imaging modality in patients with suspected appendicitis due to several factors, including lack of ionizing radiation, accessibility, and acceptable accuracy of diagnosis reported in the literature. However, many limitations to its use exist such as being operator-dependent, patient population (adult vs. paediatrics), and the body habitus of an individual patient. There has been a large variation in the reported sensitivity and specificity of ultrasound in appendicitis [23-25]. There is also a factor of availability of USG scans outside of normal working hours. Similar to the CT group, the patients who underwent DL had a shorter hospital stay in comparison to the group who underwent preoperative USG.

In another study [26], the use of the CT or the USG did not improve the diagnostic accuracy or decreased the negative appendectomy rate and suggests that in the atypical cases, DL should be considered which also supports our study findings.

Another point to consider is the cost of undergoing imaging (CT/USG) which in limited resources or busy hospitals could lead to an increase in the financial burden on public medical systems such as NHS in the United Kingdom.

There are a few limitations to our study which are the retrospective collection of data from a single centre experience and we did not look into the impact of the coronavirus disease 2019 pandemic on the availability of theatre, CT, and USG.

## Conclusions

This study shows that the DL is a valuable first option when trained surgeons are available for tackling RIF pain, particularly in the young age group (less than 40) where it can reduce the risk of radiation exposure, decrease LOS, and avoid complications because of perforation.
